# A bibliometric analysis of publication output in selected South American countries

**DOI:** 10.12688/f1000research.134574.1

**Published:** 2023-09-28

**Authors:** Aparna Narayan, Bharti Chogtu, Manthan Janodia, Raghu Radhakrishnan, Santhosh K. Venkata

**Affiliations:** 1 Manipal College of Dental Sciences, Manipal Academy of Higher Education, Manipal, Karnataka, India; 2 Kasturba Medical College, Manipal Academy of Higher Education, Manipal, Karnataka, India; 3 Manipal College of Pharmaceutical Science, Manipal Academy of Higher Education, Manipal, Karnataka, India; 4 Manipal Institute of Technology, Manipal Academy of Higher Education, Manipal, Karnataka, India

**Keywords:** South America, Publications, Citation, Research, GDP, FWCI

## Abstract

Research output provides an insight into the development of the scientific capability of a country. Budget allocation for research and development (R&D) is directly proportional to the research output of a country. Bibliometric analysis of South American countries has not been done in many studies. The purpose of this paper was to analyse research outputs from South American countries on various metrics. An analysis was done for a period of 11 years from 2010 to 2020. The analysis revealed that Brazil with highest percentage of research spend has lowest Field Weighted Citation Impact (FWCI). This contrasts with Uruguay, whose FWCI is high despite comparatively lower spend on R&D and lower publication output. Although Argentina has the highest percentage of researchers per million population (1202), it has the least papers per researchers (0.3 per year) among the countries studied. A huge disparity in terms of percentage of research spent, research output, papers per researcher, and output with national and international co-authorship was observed.

## Introduction

The research output of any country is an important indicator of its progress and knowledge development. It is imperative to assess the priority given to improving the R&D culture among the scientific community. Scientists in most countries receive varying proportions of funds from numerous sources and funding agencies mandate that the outcomes of the research is shared globally and has a societal impact. Dissemination of research transpires through publication in scientific journals which have high impact among the scientific community. Research through collaborations – institutional, national, and international – lead to cross pollination of ideas and magnifies research visibility. The USA is traditionally considered to be a hotspot of academic research. Mervis (2017) has reported that since 2012, the share of basic research funding in USA has decreased whereas corporate funding has significantly increased.

According to World Bank data, research expenditure as a percentage of Gross Domestic Product (GDP) has increased from 0.57 in 2000 to 0.80 in 2015 in Latin America and the Caribbean. In the study by Zacca *et al*., 2014 have reported that the bibliometric analysis of South America’s scientific output in the field of public health is less than half the global average in terms of number of publication and citations, low visibility and fewer collaborations are cited as the reasons of the same. Ciocca & Delgado (2017) have discussed the limitations that research faces in South American countries including an insufficient budget, poor infrastructure, the high cost of scientific material, and other uncertainty among scientists. In a recent study presented by ( Estenssoro *et al*., 2016) Brazil proposed strategies to freeze of their research budget up to 42% of the total budget in 2019. A study by Angelo (2019) reported that international collaboration is prudent in all sectors of production, including scientific output, and plays an important role in magnifying research visibility. Brazil, Mexico, Chile, and Argentina are the main countries in South America with high research outputs. One of the challenges faced by researchers is their lack of fluency in the English language, which makes it difficult for them to collaborate with researchers in the USA and Europe ( Coronel *et al*., 2011). Notwithstanding these shortcomings, a study by Packer and Meneghini (2006) for Latin American and Caribbean Centre on Health Sciences Information (BIREME)/Pan American Health Organization (PAH O)/World Health Organisation observed that articles published by authors from Brazilian institutions with more than 100 citations, received more citations for papers published with international collaborations (84.3%) than Brazilian institutions alone (15.7%). Glänzel *et al*., (2006) and Koljatic & Silva (2001) have provided scientific output from Brazil and South American countries in the fields of economics and business administration. Hermes-Lima *et al*., (2007) noted that South America’s research publications increased from 1.8% in 1991-1995 to 3.4% during 1999-2003.

Scientific and technological policies in Argentina have aimed to create knowledge to solve local issues such as social, productive, and environmental problems. A study by Miguel *et al*., 2015 found that more than 25% of research output in Argentina addressed local topics of interest, including social sciences, economy, management, agricultural and biological sciences, earth and planetary and environmental sciences, published mostly in foreign journals. In recent years, owing to geopolitical instability, research in Argentina has suffered due to financial austerity by the government which led to a reduction in science funding by 40% between 2015 and 2018 ( Catanzaro, 2018). In the study by Cross *et al*., 2017 have reported that globally, Brazil ranked thirteenth in terms of research publications produced between 2011 and 2016 and is the only South American country to feature among the top 20 countries publishing most research papers worldwide, spending approximately USD 20 billion (79.2 billion reais) on research in 2016 ( Faleiros, 2018). Chile has seen growing anger among its scientific community due to the lack of government funding ( Bajak, 2017). Colombia, on the other hand, proposed the creation of a Ministry of Science, Technology, and Innovation in 2018 to address concerns about research funding ( Bajak, 2018), which was voted for by the country’s senate. This move is expected to improve scientific priorities in the country ( Wight, 2019). Study by Jose and Michael, 2018 have reported that Ecuador’s government has implemented policies to boost scientific productivity in recent years, with the country’s research productivity improving 5.16 times more between 2006 – 2015.

Bibliometric analysis of publication outcomes of authors from Latin America publishing during the period 1989 to 2018, in topics related to epilepsy, is reported by Morán-Mariños *et al*., 2020, the published paper describes the scholarly output and citations outcomes of those publications during the period 1989 to 2018. A study by Alarcon-Ruiz *et al*. in 2019 reported that publication outcomes of Latin American countries in the form of scholarly outcomes, citations, collaborations, etc., are presented for the period 2003 to 2017 in stokes topic, it was seen from the published data that the publication number and collaboration has been increasing and citation numbers are following the increment with the lower rate. A detailed presentation of publication data related to the subject area of Energy for the Latin American region is reported by Araújo and Costa, 2016. This work presented the details related to publication outcome from Latin American countries in the subject area of Energy. Bibliometric analysis in terms of number of publications, citation in the subject area of health reforms ( Macías-Chapula, 2002) and Economics ( Bonilla *et al*., 2015) is discussed in detail. Analysis of scientific research in the subject of physics at the institutional level of Latin America is reported by Luna-Morales *et al*., 2022), the study showed that over the years the publication outcomes in terms of numbers and citations are constantly increasing. Bibliometric and scientometric analysis of scholarly publication in the subject area of Science and Technology in Latin America is reported by Gonzalez-Brambila (2021) study involves understanding the publications number, citations and institutional breakup.

A detailed study of LATAM publications in the areas of innovation in business, management, and accounting for the period 1983 to 2018 ( Cortés-Sánchez, 2019) showed that Brazil and Colombia had more cited work compared to other countries in the region. Furthermore, it was seen that those publications co-authored with non-LATAM authors had the highest impact in terms of citations and h-index. A bibliometric study by Bonilla *et al*. (2015) on the quantitative output of publications in the field of economics from Latin American countries using the Web of Science database for the period 1994 to 2013 showed that Brazil, Mexico, Chile, Argentina and Columbia have significant numbers of publications in the area of economics. It was also seen that publication numbers have been increasing significantly over the last five years. Bibliometric analysis of research on health inequalities was carried out in Latin American and Caribbean countries ( Almeida-Filho *et al*., 2003) using the indexed publication published between 1971 and 2000. The results reported publication growth in countries like Brazil, Chile, and Mexico. Analysis of publications indexed in the Scopus and Web of Science databases between 1987 to 2016 ( Tello Gamarra *et al*., 2018) showed that growth in publications over this period in Latin America was not significant, relative to other parts of the world.

Recently, there have been many collaborative studies with countries like the United States, Spain, and the United Kingdom. Analysis of the Scopus database under the category “Public Health, Environmental and Occupational Health” for the period 1996-2011 in the region of Latin America ( Zacca-González *et al*., 2014) presented this in terms of citation, publication numbers, co-authors, and collaborations. A study reported by Teixeira, 2014 provided descriptions of the evolution, function, and influence of the National Systems of Innovations in these Latin countries and analyzed the literature published during the period 2006-2010. Analysis of publications from 2000-2017 in Social Science Citation indexed journals on the theme of corporate social responsibility in Latin America was presented by Jaén *et al*., 2018 and helped in identifying several key areas of industrial concentration in the Latin American region. A bibliometric study of scientific journals to understand the influence of internationalization in science by ( Zitt & Bassecoulard, 2004). Capobianco *et al*., 2019 reported the use of Scopus indexed publications from the period 1983-2017 for the analysis, to show the effect on publication quality and other metrics in the context of national and international competitiveness. Another analysis of publication impact on documents published during the period of 2002 to 2016 in Web of Science indexed journals was presented by Ahmad *et al*., 2018. This presented a detailed discussion of citation analysis, the profiles of journals, the countries contributing, the type of documents published and subject in which the journal is publication. Though material sciences are a growing research field in Uruguay, they are still underrepresented as compared to global average ( Aguiar *et al*., 2018). Given its share of the world population and GDP, publications from South America are still fewer than what might be expected ( Van Noorden, 2014). Elango *et al*., 2021 report analyses the publication outcome in terms of scholarly output and citations from India and South Korea. Islam and Roy, 2021, presented the improved research productivity in terms of publication numbers from Bangladesh during the period 1971-2020.

Despite the challenges, South American countries have witnessed an economic growth in the last two decades leading to enhanced investments in R&D, eventually translating into increased publications from research, few studies have attempted to understand the scientific publication landscape of South America in recent years as certain changes have occurred. There is a need to understand the change in the research metrics with changes happening at the global level. The research analysis of Latin American countries will give us an insight into the publications, citations, FWCI, research spent and international collaborations. From the background study reported by different researchers it is clear that the bibliometric study is important to understand the publication phenomena across the country, this data can be further used for strategizing or policy formation at the country level.

With this background, this paper aimed at analyzing the research output of South American countries and correlating the percentage of research spend to research output and to research output per million population, Field Weighted Citation Impact (FWCI), papers per researcher, and research output within different subject categories. The study aims at comparing these metrics between the countries in this region. The correlation between R&D spent and research outcomes have been analyzed and how these numbers are changing over the years. Also this article focuses on how the different countries in this region work in different research areas and the impact of internationalization on research quality. Overall, this study aims to summarize the research contributions of seven major South American countries based on their overall research contribution.

## Methods

### Data source

Data were extracted from one of the largest abstract and citation databases of peer-reviewed literature; Scopus and its affiliate SciVal. The ranking agencies, Quacquarelli Symonds (QS) and Times Higher Education (THE) provide global and regional ranking of universities based on scientific output in these citation databases. Furthermore, Scopus and SciVal provide options to retrieve various scientific parameters through certain permutations and combinations for each country as well as for the region. All types of articles, such as journal papers, conference proceedings, and book chapters were considered for analysis rather than concentrating solely on journal papers. Since book chapters and conference proceedings can also be cited, these article categories were retained for analysis. Publication analysis was carried out in terms of trends in publication, citation, relation between publication outcomes across LATAM countries, subject wise trend analysis of publication for a period of eleven years from 2010 through 2020.

The search string with regard to publication during the period 2010 to 2020 is used to understand the publication trends, citation trends, collaboration trends, quality publications trends across various subject areas like Arts & Humanities, Engineering & Technology, Life Sciences & Medicine, Natural Sciences and Social Sciences.

World Bank Indicators for identifying research expenditure as a percentage of GDP, researchers per million population, and total scientific output were used. The latest data related to research expenditure by the countries, population data, researcher population data are extracted from the available world Bank indicators were captured for the analysis.

*Selection of countries for analysis*

Seven countries were selected for analysis based on certain parameters, such as total population, research output in a given year, research expenditure as a percentage of GDP spent by the countries, and publications with national and international collaborations. Based on predefined criteria, Argentina, Brazil, Chile, Colombia, Ecuador, Mexico, and Uruguay were selected. Venezuela and Cuba were excluded due to the current political situations in those countries. The remaining South American countries were excluded as they did not meet the predefined criteria above.

### Search strategies

*Research spend versus research output*

The analysis of scientific disciplines was based on subject categories. Publications are divided into different subject categories in Scopus. Around 300 subject categories are used within the five major subject areas namely Art and Humanities, Engineering and Technology, Life Science and Medicine, Physical Science and Social Science and Management. The subject categories were classified based on the QS and THE, however, several similar categories (with name variations) were combined for ease of analysis. QS provides five subject categories, while THE provides for eleven subjects. The five major subject areas referred to above were used for carrying out analysis of publication of in terms of number of publication, citation received during the period of study, international collaboration publications and its output, publications in the top quartile journals.

### Findings

*Selection of countries for analysis*

It could be deduced from this analysis that Brazil has the highest percentage of research spend and the lowest Field Weighted Citation Impact (FWCI) among the South American countries studied. Contrary to that, Uruguay has a lower percentage of research spend than all other countries, except Colombia, but it exhibited the highest FWCI. Uruguay has around 645 researchers per million population according to World Bank data compared to 900 researchers per million population for Brazil.

**Figure 1.**
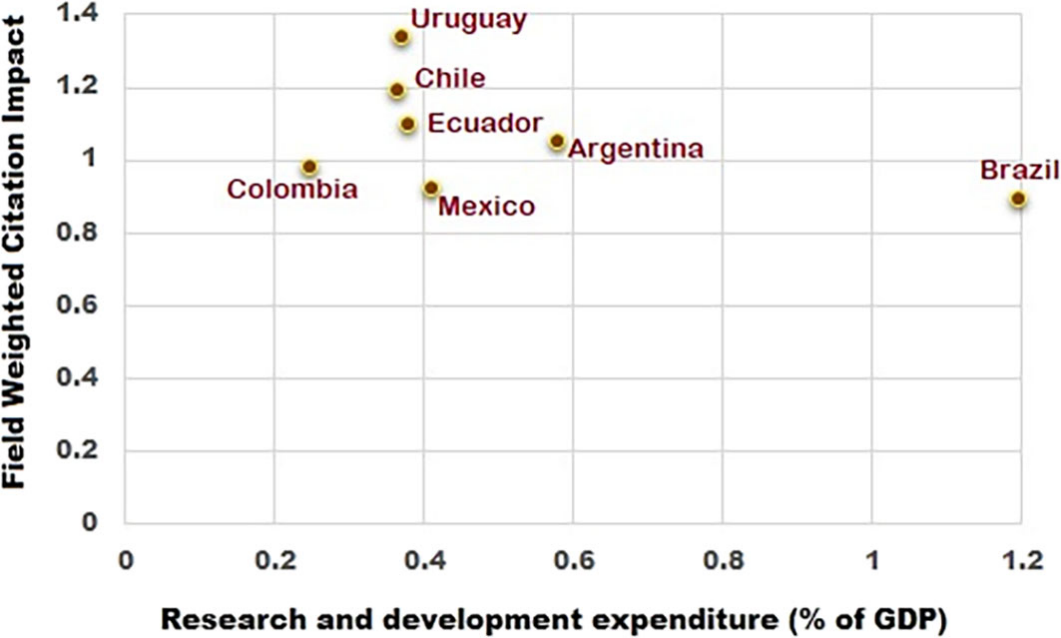
Percentage of research spent vs field weighted citation impact (FWCI) for the period 2010 to 2020.

**Figure 2.**
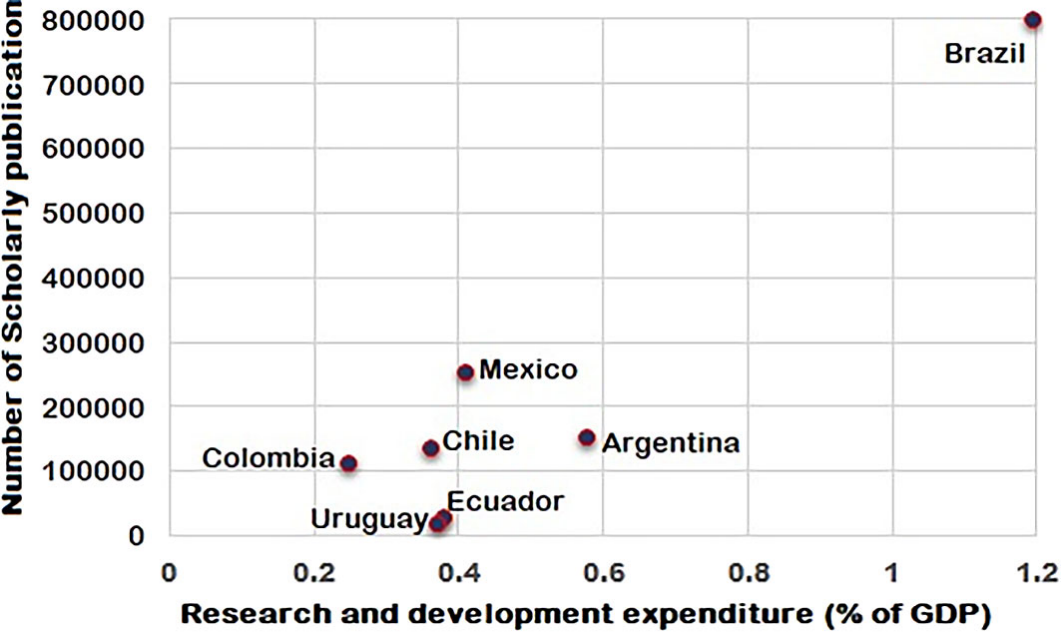
Percentage of research spent vs scholarly output for the period 2010 to 2020.

**Figure 3.**
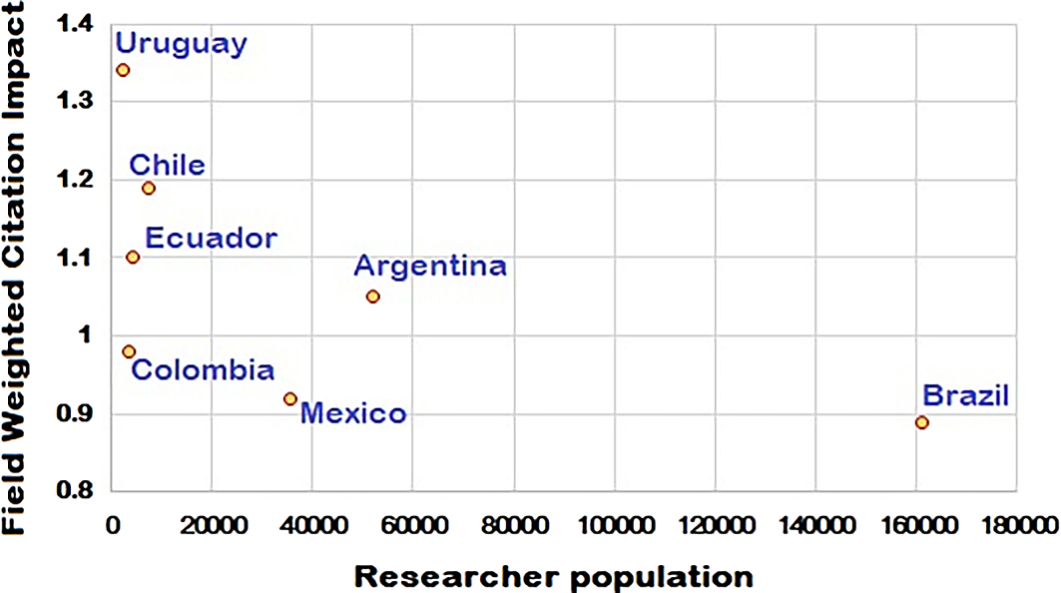
Field weighted citation impact (FWCI) vs total number of researchers for the period 2010 – 2020.

This shows that despite fewer researchers per million population, the FWCI of Uruguay is significantly better. From the Figure 1, Figure 2 and Figure 3, it is clear that the difference in the total number of scientific articles published from Brazil and Uruguay is great. In 2014, Brazil collectively published 53,606 articles compared to a total of 809 articles published by Uruguay. R&D expenditure as a percentage of GDP for Brazil in 2015 was 1.27%, compared to 0.36% for Uruguay. Chile spent 0.36% (2016), Colombia 0.27% (2016), and Ecuador 0.44% (2014) of their GDP on R&D. The percentages of GDP spent on R&D by Argentina and Mexico were 0.63% (2015) and 0.50% (2016) respectively, as shown in Figure 1, which were higher than many other Latin American countries, aside from Brazil. The amount spent on research by Brazil is quantitatively proportional to output in terms of the total number of research publications, but it is inversely proportional to FWCI. This contrasts with Uruguay, whose FWCI is high despite comparatively lower spend on R&D and lower publication output. The research outputs of Argentina and Mexico are proportional to the percentage of research spend as shown in Figure 2. Chile has 502 researchers per million population, Colombia has 131, Uruguay 645, and Mexico 245.

*Number of researchers versus quality of publications*

**Figure 4.**
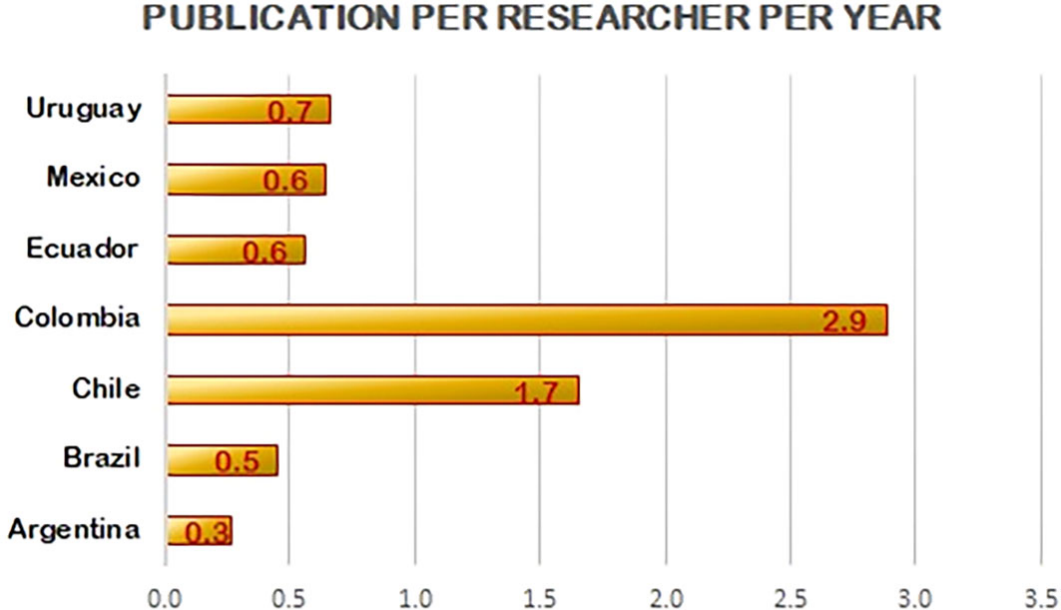
Scholarly output per researcher (in total publications) for the period 2010-20.

As seen in Figure 3, among the countries studied, despite having the highest number of researchers per million population (1202), Argentina has the least papers per researcher (0.3 per year). Although R&D spend by Colombia is lower and there are less researchers per million population, the figures for papers per researcher is 2.9 per year, which is the highest of all the countries studied. This is followed by Chile (1.7 per year) even though it has the lowest percentage of GDP spent on research. Brazil has the highest percentage of research spend and 0.5 papers per researcher per year. Uruguay and Mexico have 0.7 and 0.6 papers per researcher per year, respectively. Research output is not proportional to the percentage of GDP spent on research as represented in Figure 4. From the data projected in Figure 4 expenditure on research is not directly propositional to the output of research by the country. Research spending should be combined with strategies to help increase output per researcher by other means such as collaborations that emphasize publishing in high quality journals.

National collaborations have accounted for an improved FWCI for Uruguay whereas it has not led to improved FWCI for Brazil. Uruguay, with 650 researchers per million population, and a 0.44% GDP spend on R&D, achieved a higher FWCI than Colombia, Chile, Mexico, and Argentina. Carefully crafted national collaborations are an important parameter for improved research output as well as research visibility. Uruguay, Ecuador, and Chile have better FWCI with international collaborations. Uruguay has a better FWCI than Ecuador, which has a smaller percentage of international collaboration although this is greater than that of Chile. Notably, Brazil has the least international collaboration leading to the worst FWCI of the countries. The data show a direct correlation between international collaboration and FWCI.

*Impact of collaborations on research output*

**Figure 5.**
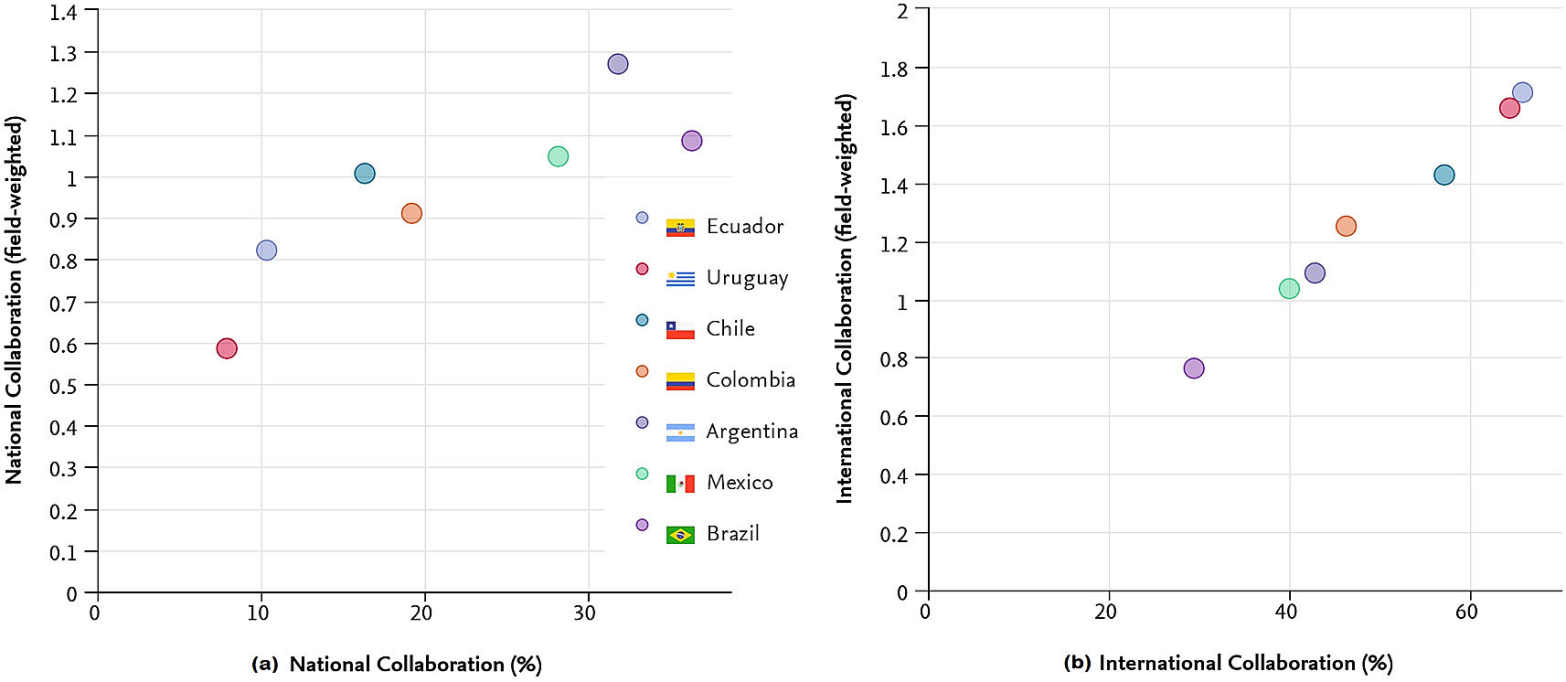
Collaboration vs field weighted citation impact for the period 2010-20 (a) National (b) International.

In this study, the analysis aims to understand the impact of collaboration as a signifier of research outreach. It has been observed that Brazil has the highest quantum of publications with national collaborations (36.3%), followed by Argentina (31.8%), Mexico (28.2%), Colombia (19.2%), Chile (16.3%), Ecuador (10.3%) and finally Uruguay (7.9%), having the least number of national collaborations is shown in Figure 5a.

International collaboration refers to publications with at least one author having an affiliation outside the home country. The impact of this collaboration is analyzed and represented in Figure 5b. It is apparent that during 2010-20, Ecuador produced the largest percentage of publications with international co-authorship (65.7%), followed by Uruguay (64.4%), Chile (57.1%), Colombia (46.2%), Argentina (42.8%) and Mexico (40%). Brazil (29.5%) had the least number of international co-authored publications.

To understand the impact of these publications, the FWCI was compared with the collaboration. Although Brazil has the highest percentage of national collaborations, it has the least international collaborations and the lowest FWCI (0.76) in comparison to all other countries. Ecuador has the highest FWCI (1.71) with greater international collaboration at around 65.7%. Uruguay with international collaborative work of 64.4% and an FWCI around 1.66. It was also noted that the countries with higher international collaboration have higher citation impact.

*Citation impact of South American research output*

**Figure 6.**
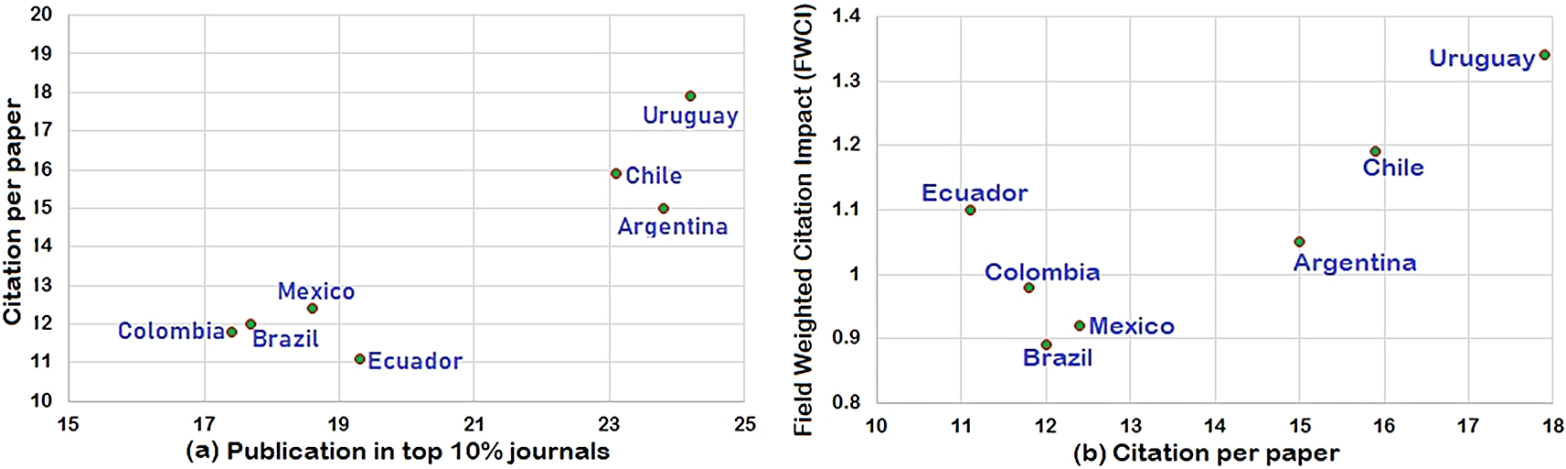
(a) Outcome in top 10% journal with citation per paper b) field weighted citation impact (FWCI) vs. citation per paper.

Citation impact of the South American countries is analyzed in Figure 6(a) and Figure 6(b). Figure 6(a) compares publication output in the top 10% of journals versus citations per paper, which reflects the quality of publication. Publication from Uruguay has the highest citations per paper of 17.9, while citation per paper for Ecuador has lowest c/p at 11.1; publication from Argentina has citations per paper of 15. Publication from Chile has 15.9 citations per paper compared to 12.4 for Mexico. Figure 6(b) represents citations per paper with FWCI for each country. Uruguay has the highest citations per paper (17.9) with the highest FWCI of 1.34; Ecuador with the least citations per paper 11.1 has FWCI of 1.1. Countries like Mexico, Argentina, and Chile follow a similar trend – Mexico with 12.4 citations per paper has 0.92 FWCI, Argentina has 15 citations per paper with 1.05 FWCI, and Chile has 15.9 citations per paper with 1.19 FWCI. Colombia and Mexico show a different trend; Colombia has 11.8 citations per paper and a FWCI of 0.98 and Ecuador has 12.4 citations per paper with a FWCI of 1.1. These details clearly establish the direct correlation between FWCI and citations per paper, i.e., the higher the citation per paper, the greater the FWCI.

*Subject area analysis*

Table 1 shows the outcomes of countries in the subject areas for the period 2010-20. The majority of the publications (35-50%) are in the subject areas of Life Sciences and Medicine, with a similar trend seen for all countries studied. The least number of publications is in the Arts and Humanities, which is around 1% to 5% across the LATAM countries. Ecuador and Colombia produce more papers in Engineering and Technology whereas Argentina, Chile, Mexico, and Uruguay produce more publications in Physical Sciences. Brazil’s outcome is almost similar in both these subject areas. Social Science and Management publications are the fourth largest among all countries, with a similar share of publications (around 10-12%). Chile produces a large percentage of Arts and Humanities publications while Brazil produces the least. Similarly, Uruguay produces a large percentage of Life Sciences and Medicine publications, and Chile has the least. It is of note that South America has about 45% of publications in the subject category Life Science and Medicine against a global trend of 35% whereas the total publication output in the subject category Engineering and Technology is less in South America (20%) compared to global figures (28%).

**Table 1.** Classification of publications across subject areas during 2010-2020.

Name	Arts & Humanities	Engineering & Technology	Life Sciences & Medicine	Physical Science	Social Sciences & Management
**Argentina**	9934	30397	82687	49302	20858
**Brazil**	21442	207232	451714	199181	108446
**Chile**	10592	29943	57041	45503	25973
**Colombia**	6264	36115	48913	28694	22121
**Ecuador**	825	11321	11137	7071	6064
**Mexico**	8431	79804	118291	85963	34582
**Uruguay**	541	3757	9572	4138	2384
**South America**	50777	319499	660404	323377	189067
**Global**	1740442	12028256	14051299	9829793	5116621

**Figure 7.**
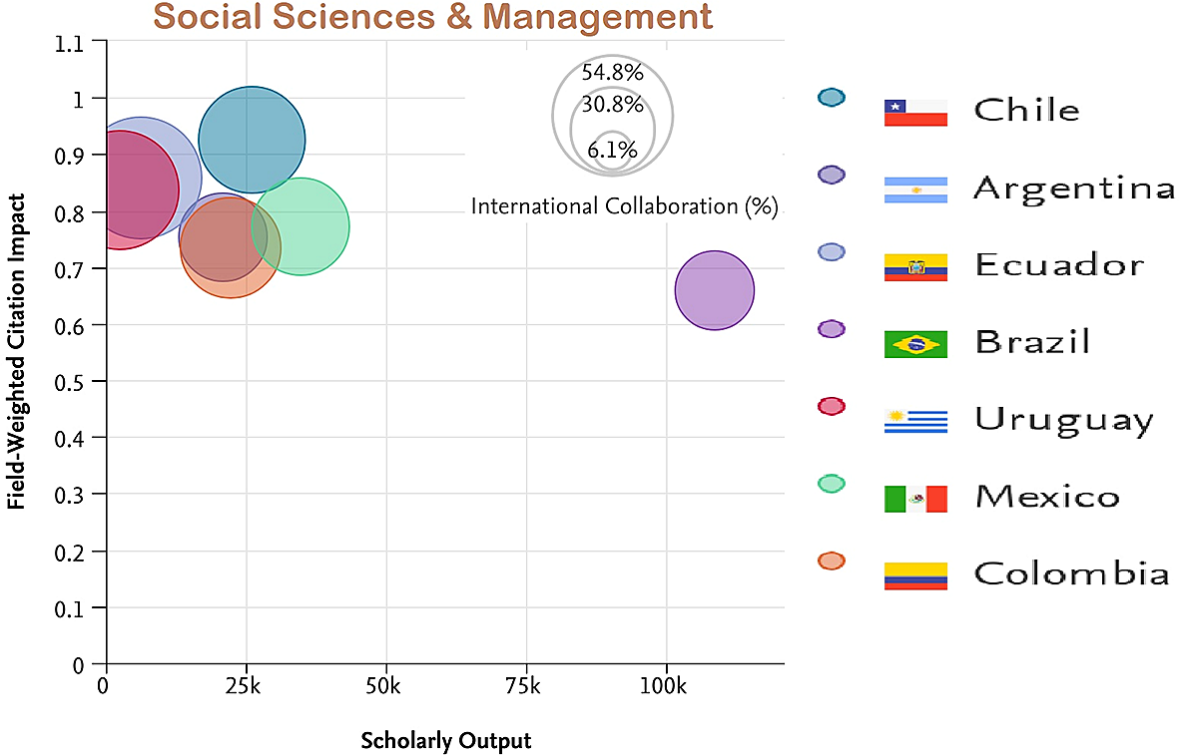
Field weighted citation impact vs scholarly output vs international collaboration in Social science & Management.

Figure 7 to Figure 11 depict the percentage of international collaboration by the size of the bubble (i.e., the larger the bubble, the more international collaboration). The countries subjected to analysis show a better FWCI in the subject areas of Life Sciences and Medicine as compared to Arts and Humanities which has the lowest FWCI.

**Figure 8.**
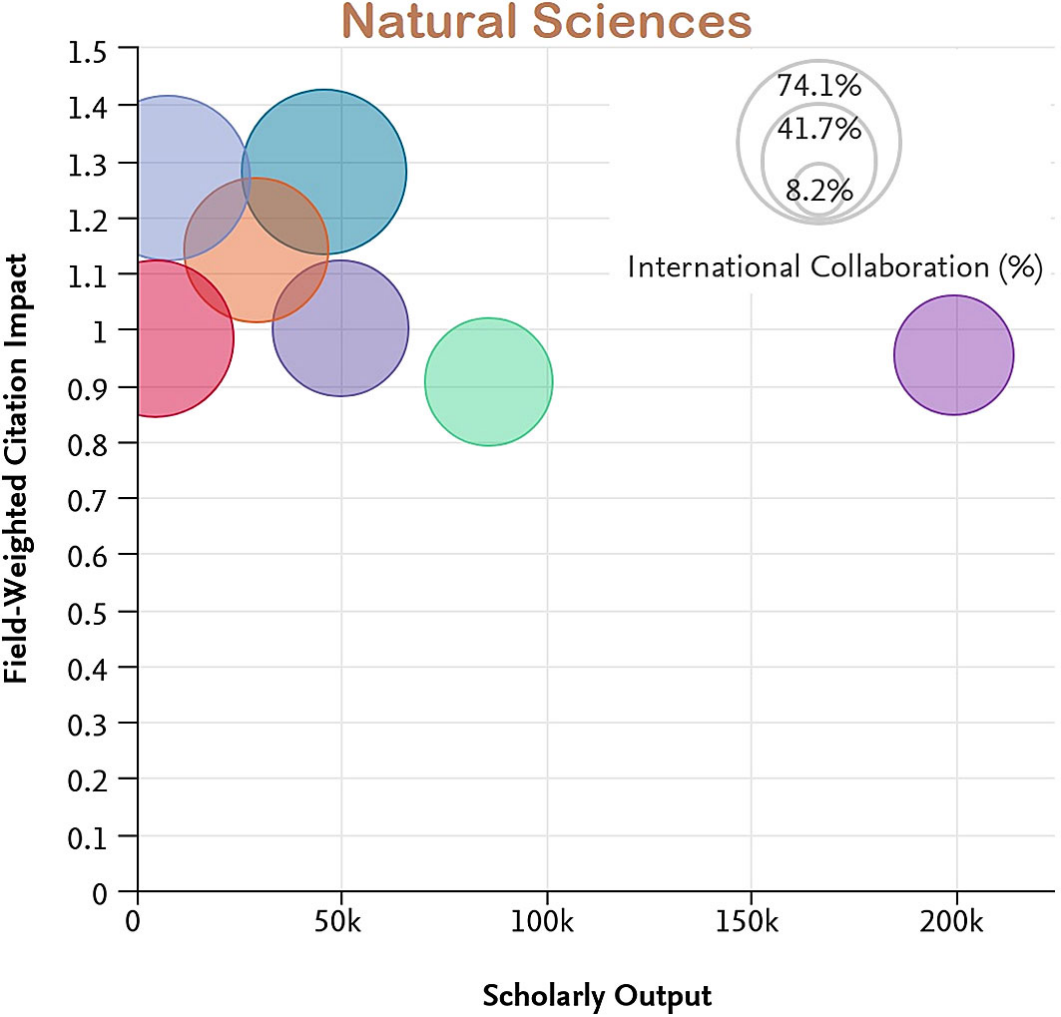
Field weighted citation impact vs scholarly output vs international collaboration in Natural Sciences.

**Figure 9.**
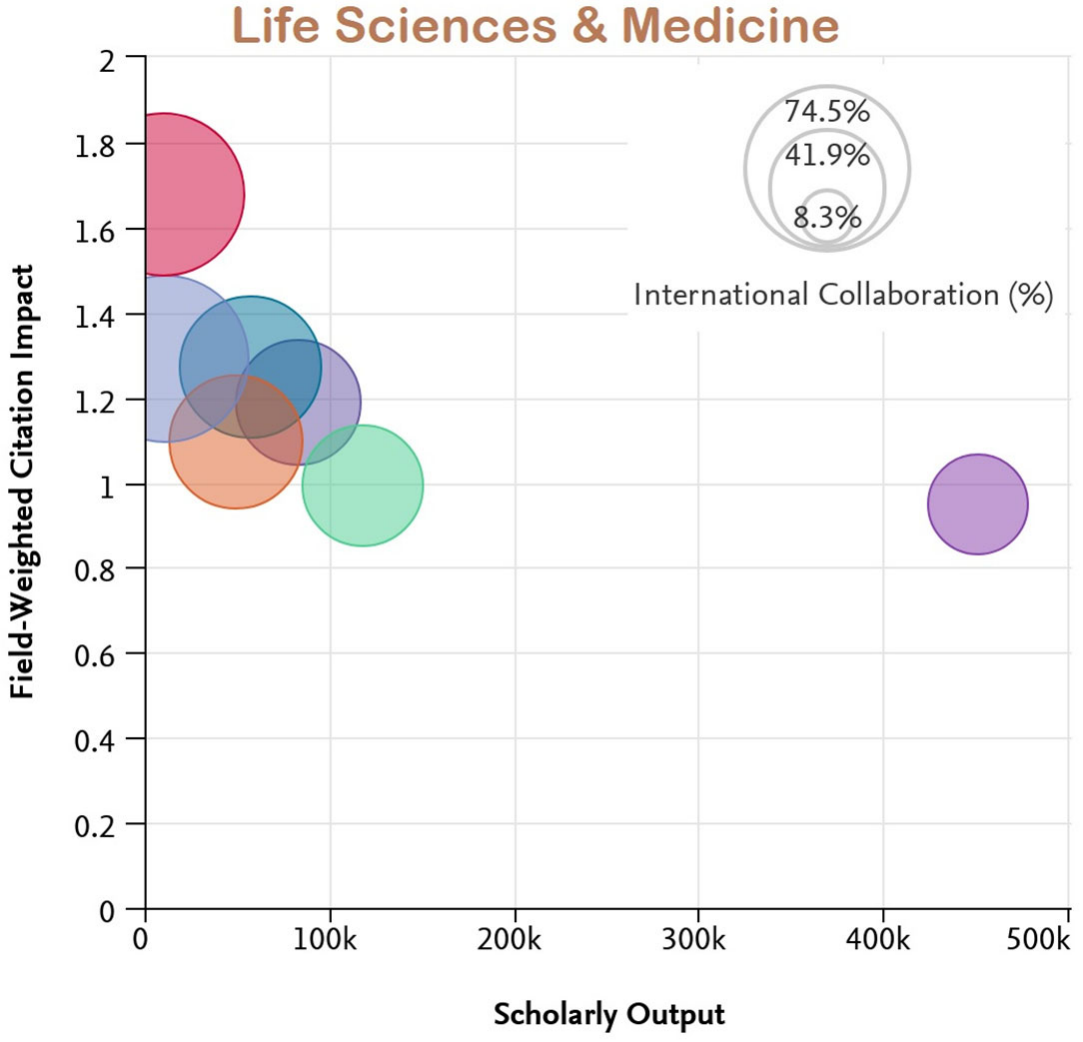
Field weighted citation impact vs scholarly output vs international collaboration in Life Sciences & Medicine.

**Figure 10.**
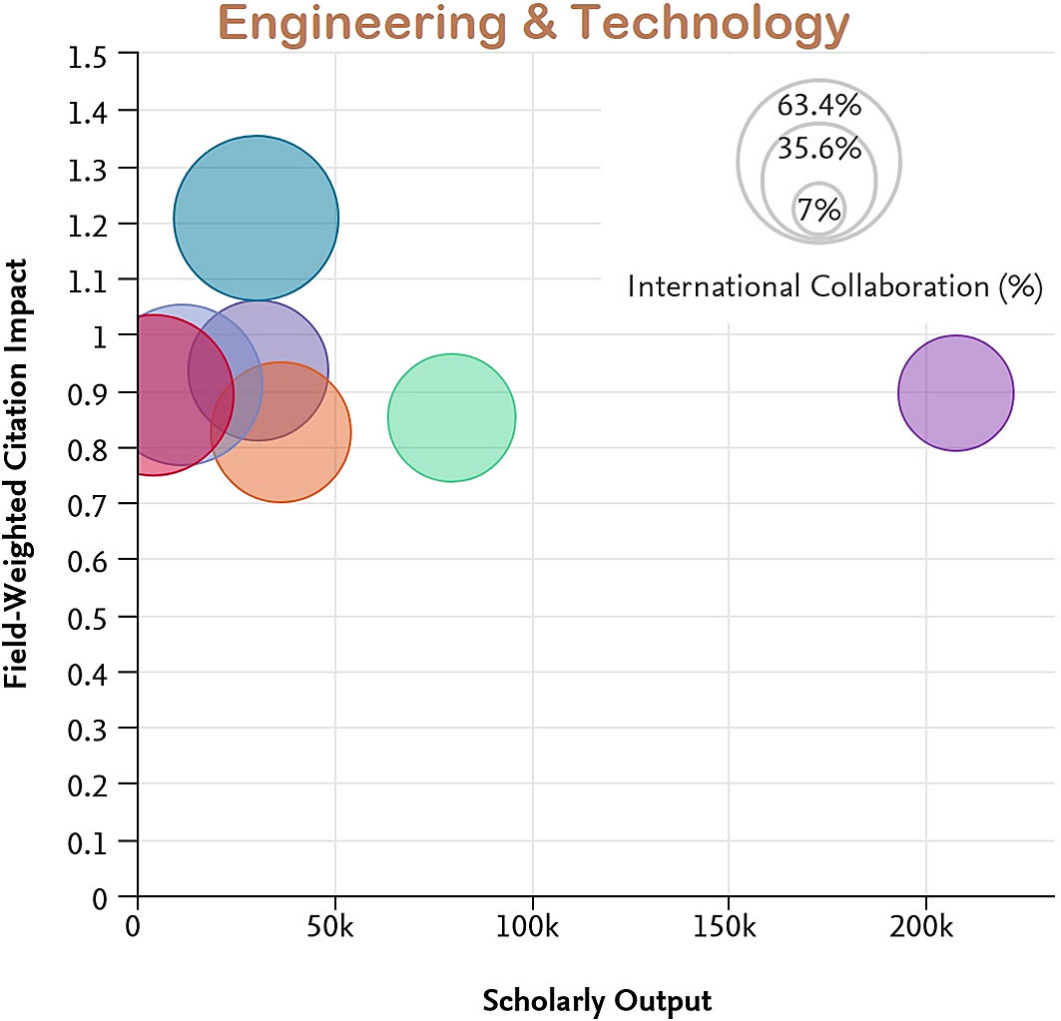
Field weighted citation impact vs scholarly output vs international collaboration in Engineering & Technology.

**Figure 11.**
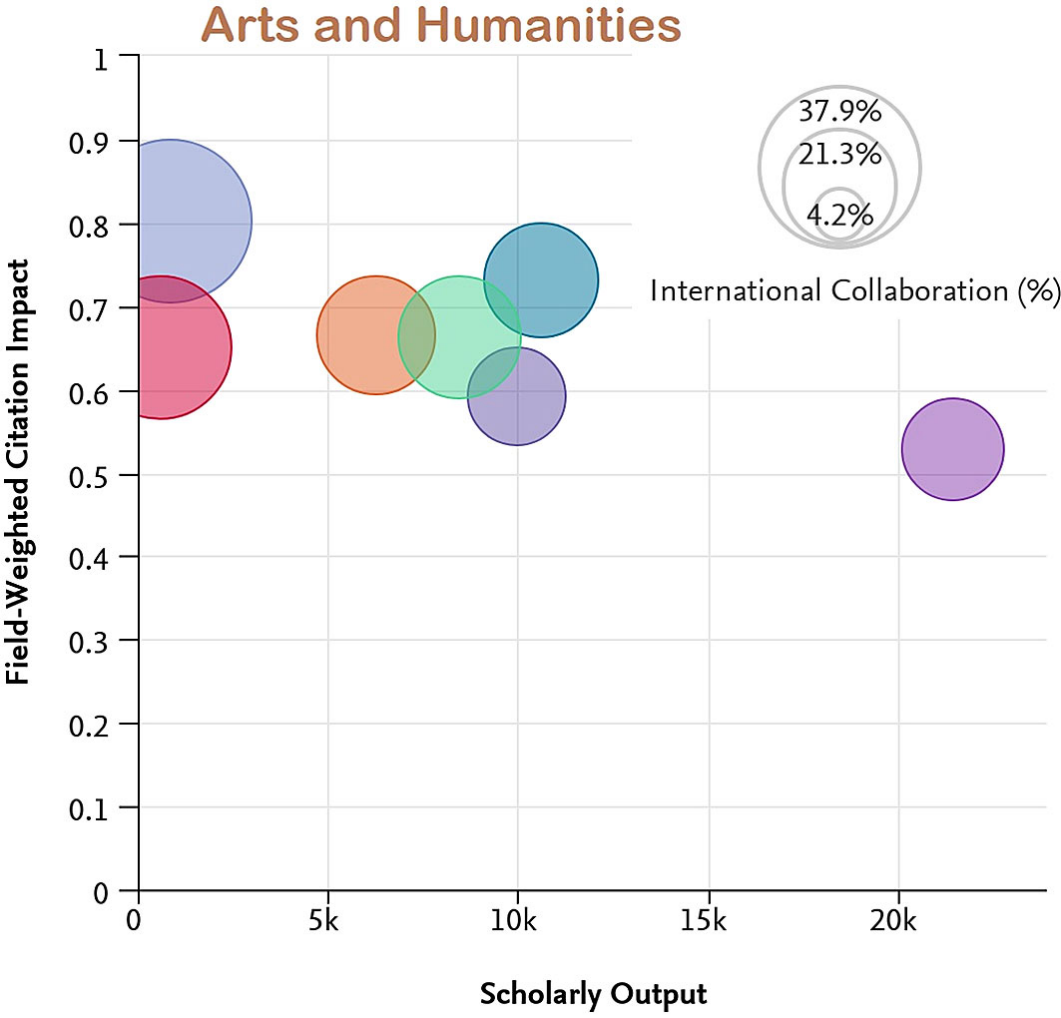
Field weighted citation impact vs scholarly output vs international collaboration in Arts and Humanities.

Among the subject categories, Ecuador’s FWCI is highest in Arts, despite lower scholarly output. A plausible explanation is that this is due to the high number of publications through international collaboration. Brazil has the least FWCI in the subject category of Arts, which is lower than the average across Latin America. Uruguay’s FWCI is less than Chile, Colombia, Mexico, and Argentina. However, the FWCI of these four countries in the subject category Arts is better than the average for the region. Similarly, for the subject category Engineering and Technology, Ecuador and Chile have higher FWCI despite lower scholarly output compared to Brazil, Mexico, Columbia, and Argentina. Uruguay’s performance is better in the subject category Life Science and Medicine, and is followed by Ecuador, Chile, Argentina, Colombia, and Mexico. Brazil appears last in the list. Ecuador, Chile, Colombia, and Argentina perform better than Brazil, Uruguay, and Mexico in the subject category Physical Sciences. In Social Science, Chile has the highest FWCI compared to Ecuador, Uruguay, Mexico, Argentina, and Brazil. Notable performance for Ecuador, Uruguay and Chile could be attributed to their improved international collaborations compared to their counterparts.

## Conclusion

The research outcomes of a region provide an insight into the focus on knowledge building and working towards societal challenges. These metrics provide information about the quality of higher education and the amount of research spent which further gives an idea about the commitment of government towards progression of science and development. Bibliometrics is one of the ways in which research outcomes can be quantified. Bibliometric data includes information related to number of publications, quality of publication, which is a direct indicator for the research spent, human resource available, government policies on research and development. One of the metrics explored in this analysis is collaborative publication which is an indicator of how the authors and researchers are open to collaborate with different countries and target global challenges in addition to their local and national challenges.

In the work presented a detailed bibliometric study of south American countries (LATAM countries) namely Argentina, Brazil, Chile, Colombia, Ecuador, Mexico and Uruguay has been done. A detailed analysis was presented about the publication outcome of all the above countries during the period 2010 to 2020 in Scopus indexed journals. The study also presented a detailed analysis of the research outcome in terms of scholarly output, citations per publication, Field Weighted Citation Impact, Citation count. An analysis about the collaboration of researchers across the globe is presented with reference to collaborative publications. The publication outcomes were also analyzed based on the journal subject area which gives us an understanding of the research focus areas in this region. A huge disparity in terms of percentage of research spend, research output, papers per researcher, and output with national and international authors has been found. Higher research spent can improve the quality of publications and thereby citations but paradoxically a positive correlation has not been observed between research spent and increased FWCI. Brazil, with higher research spend, has lower research productivity while Chile, with lower spend on research has the highest figures for papers per researcher. Ecuador has the highest percentage of publications with international co-authorship while Uruguay has the highest FWCI and higher international collaborations. Chile produces a large percentage of Arts and Humanities publications whilst Uruguay produces a large percentage in Life Sciences and Medicine. FWCI of Ecuador is highest in Arts despite low scholarly output. As a global trend in this region also international collaborations have led to an increase in FWCI and greater research impact.

The countries need to focus on prominent areas of research and collaborate with other countries to increase the visibility of research. Individual nations should recognize their research expertise and work with researchers across the globe which applies more to small countries with low economies where the research spent is low.

## Data Availability

Open Science Framework: LATAM https://doi.org/10.17605/OSF.IO/FU3QC ( Santhosh *et al*., 2023) This project contains the following underlying data:
• Citation_top10.xlsx (description of file) •
FWCI_Citation_National.xlsx: Citation data of all the LATAM countries • FWCI_publicationoutput.xlsx: FWCI data compared with publication of LATAM countries •
FWCI_citation_Scholary.xlsx: FWCI data compared with publication and citation of LATAM countries •
FWCI_Publicationoutcome_Arts.xlsx: FWCI data compared with publication and citation of LATAM countries in arts & Humanities subject area •
FWCI_publicationoutcome_Eng.xlsx: FWCI data compared with publication and citation of LATAM countries in Engineering & Technology subject area •
FWCI_publicationoutcome_life.xlsx: FWCI data compared with publication and citation of LATAM countries in Life Science & Medicine subject area •
FWCI_Publicationoutcome_Natural.xlsx: FWCI data compared with publication and citation of LATAM countries in Natural Science subject area •
FWCI_publicationoutcome_Social.xlsx: FWCI data compared with publication and citation of LATAM countries in Social Science subject area • NationalCollaboration_FWCI.xlsx: FWCI data compared with National collaboration of LATAM countries. • Internationalcollaboration_FWCI.xlsx: FWCI data compared with international collaboration of LATAM countries • working.xlsx: LATAM bibliometric data Data are available under the terms of the Creative Commons Zero “No rights reserved” data waiver (CC0 1.0 Universal).
