# Supplementary material for: A bibliometric analysis of publication output in selected South American countries
Source: F1000Res. 2023 Sep 28;12:1239. [Version 1] doi: 10.12688/f1000research.134574.1 (PMC10696296; doi:10.12688/f1000research.134574.1)
Supplement: Peer review history

# Reviewer report: Reviewer response for version 1

**DOI:** 10.5256/f1000research.147639.r212600

**Published:** 2024-04-12

**Authors:** Nabi Hasan, Manu T R

**Affiliations:** 1 Central Library, Indian Institute of Technology Delhi, New Delhi, Delhi, India; 2 Indian Institute of Technology Delhi, New Delhi, Delhi, India

**Summary of the article**

 The researchers have undertaken a study of bibliometric analysis of publications of selected LATAM countries to identify their research growth and analyzed with their research funding/spent, population growth/ researcher population of selected countries. The researchers identified the subject-wise trend analysis of publication for eleven years from 2010 through 2020. The SciVal, a Scopus sources were used to retrieve the required data for this study. In findings, researchers shown percentage research spent vs field weighted citation impact (FWCI), percentage research spent vs scholarly output, scholarly output per researcher, international collaboration, collaboration vs. weighted citation impact, citation impact of South American research output, subject area analysis, etc. to fulfill the mentioned objectives. It will benefit researchers, policymakers, and funders to analyze ROI in scholarly research output of these countries and focus on the less-funded research areas. In this manuscript, the researchers may consider addressing the following to make it more significant:

**Abstract**

- Data sources for support data can be specified in the abstract to make it clear and comprehensive from the beginning.

 
**Introduction  **

- Adding the highlights on current expenditure on R&D and of the selected countries’ would add more value to this article. Growth of expenditure on R&D over a period from 2010 to 2020

**Methodology**

- A clear definition of Field Weighted Citation Impact (FWCI) and weightage of different disciplines may be required in the methodology. 
- It states that the latest data related to research expenditure by the countries, population data, and researcher population data are extracted, but the researchers have analyzed publications from 2010 to 2020. Not recent publications have been considered. Would it be correct to consider comparing the research expenditure or GDP of a particular year and the publication of the same year because research expenditure or GDP is also increased over the period? The same case can be applicable to compare the researcher population. 

**Cited References**

- Most cited references are from prior to 2020. Only a few recently published citations are referred to. More recently published citations are required.

Is the work clearly and accurately presented and does it cite the current literature?

Partly

If applicable, is the statistical analysis and its interpretation appropriate?

Yes

Are all the source data underlying the results available to ensure full reproducibility?

Yes

Is the study design appropriate and is the work technically sound?

Yes

Are the conclusions drawn adequately supported by the results?

Yes

Are sufficient details of methods and analysis provided to allow replication by others?

Partly

Reviewer Expertise:

Research Data Management, Scholarly Communication, Open Access, Open Science, Consortia Access Management, E-Resources Management, Science Communication, Science Mapping, Marketing of Library Resources Services and Products

We confirm that we have read this submission and believe that we have an appropriate level of expertise to confirm that it is of an acceptable scientific standard, however we have significant reservations, as outlined above.

# Reviewer report: Reviewer response for version 1

**DOI:** 10.5256/f1000research.147639.r212601

**Published:** 2023-12-04

**Authors:** Kishor Chandra Satpathy

**Affiliations:** 1 Indian Statistical Institute, Kolkata, West Bengal, India

**Abstract:** The study provides a concise bibliometric overview of the research output and scientific temper of the seven major South American countries (LATAM) based on various metric indicators during 2010–2020. The data shows notable differences between these nations on a number of indicators. In doing so, the study aims to close the knowledge gap in the less-explored areas, which may be useful for allocating resources and making numerous other policy decisions pertaining to the Latin American region.

**Introduction: **The study offers background data and a global scenario of research output measures, which serve as the main catalysts for national R&D cultures. It contains a fair overview of the relevant literature, with an emphasis on the relationship between South American countries' research output and their R&D spending. It is noteworthy that during the past 20 years, there has been economic expansion, which has raised R&D expenditures and, in turn, raised research output. As a result, the study emphasized how internationalisation affects both the volume and caliber of research conducted in this field. Though research output has increased, the analysis shows a dearth of publications that highlight the South American countries' research landscape, which justifies the rationale and scope of the study. The study also focuses on how the countries perform in various research arenas and the impact of internalization of research quality and quantity thereof. The research query is well-defined, provides insight into the publications, citations, FWCI, research expenditures, and collaboration scenarios of Latin American countries, and clearly aligns with the scope and objectives of the study.

**Methods: **This section addresses topics such as a broad summary of the data sources, search techniques, and criteria for selecting nations within the parameters of the study's aims and scope. The decision to include journal articles, conference proceedings, book chapters, and other types of documents from a variety of subject areas during the designated time period is also incorporated. The relevant figures and tables provide an elaborative representation of the findings. A thorough and perceptive study of the research output and partnership patterns of the chosen South American countries is given in the findings section.

 The use of visual representations, such as figures and tables, enhances the clarity of the presentation. The examination of research expenses versus output, the impact of collaborations on research quality, and the citation impact of South American research output add depth to the study. The identification of disparities between research spending and output, as well as the correlation between international collaboration and research impact, adds valuable insights.

**Conclusion:** It summarizes the major findings with respect to the stated research queries of the study in the context of the research landscape of the LATAM countries based on the Scopus output, and it will contribute to the existing literature.

**Comments:**

 Title: Expressive

 Abstract: Precisely presented

 Language/Presentation: Excellent

 Objectives of the Study: Specifically mentioned.

 Literature Review: Included in the introduction section

 References: Rightly appended.

**Overall remarks:**

 In general, the paper is well-written to highlight the less explored areas of LATAM countries. The findings might be used for strategizing policy formation at the country level. Overall, I see the potential for this study to contribute to the existing literature. The topic and the discussions are interesting.

**May be considered for indexing.**

Is the work clearly and accurately presented and does it cite the current literature?

Yes

If applicable, is the statistical analysis and its interpretation appropriate?

Yes

Are all the source data underlying the results available to ensure full reproducibility?

Yes

Is the study design appropriate and is the work technically sound?

Yes

Are the conclusions drawn adequately supported by the results?

Yes

Are sufficient details of methods and analysis provided to allow replication by others?

Yes

Reviewer Expertise:

Library Management, Digital Library, IR, Innovative Library Space,  Bibliometric Studies, Archival Studies, and Library Outreach Activities

I confirm that I have read this submission and believe that I have an appropriate level of expertise to confirm that it is of an acceptable scientific standard.

# Reviewer report: Reviewer response for version 1

**DOI:** 10.5256/f1000research.147639.r221512

**Published:** 2023-11-22

**Authors:** Muhammad Imran

**Affiliations:** 1 King Abdulaziz University, Jeddah, Makkah Province, Saudi Arabia

Abstract:

 The abstract discusses the correlation between research output and the scientific capability of a country, emphasizing the direct proportionality between budget allocation for research and development (R&D) and a country's research output. Notably, the study fills a gap by conducting a bibliometric analysis specifically focused on South American countries, a region less explored in previous research. The abstract hints at a significant disparity in various research metrics, including research spending, output, papers per researcher, and collaborative efforts, both nationally and internationally.

Introduction:

 The introduction provides a comprehensive overview of the significance of research output as an indicator of a country's progress and knowledge development. It effectively highlights the global importance of R&D culture, funding sources, and the dissemination of research through publications. The inclusion of key statistics, such as the World Bank data on research expenditure in Latin America and the Caribbean, adds credibility and context to the study. The reference to specific studies addressing the challenges faced by South American countries in research, funding, and international collaboration establishes a strong foundation for the subsequent analysis. The article's rationale effectively establishes the importance of understanding the scientific capability of countries through research output. The assertion that budget allocation for R&D is directly proportional to research output provides a clear justification for the study. The decision to focus on South American countries adds novelty, addressing a gap in existing literature where bibliometric analysis in this region has been limited. The research question is well-defined and aligns with the stated purpose of the paper. 

Methods:

 The methods section is clear and detailed, outlining the data sources, selection criteria for countries, and search strategies. The decision to include various types of articles, such as journal papers, conference proceedings, and book chapters, enhances the inclusivity of the analysis. The study's focus on trends in publication, citation, and collaboration across different subject areas for an 11-year period ensures a thorough and comprehensive analysis. Figures and tables are appropriate and reflect the findings in an elaborative way. 

 The findings section provides a detailed and insightful analysis of the research output and collaboration patterns of the selected South American countries. The use of visual representations, such as figures and tables, enhances the clarity of the presentation. The examination of research spend versus output, the impact of collaborations on research quality, and the citation impact of South American research output adds depth to the study. The identification of disparities between research spend and output, as well as the correlation between international collaboration and research impact, offers valuable insights.

Conclusion:

 The conclusion succinctly summarizes the key findings and emphasizes the importance of bibliometric analysis in understanding the research landscape of South American countries. The acknowledgement of disparities and the recommendation for countries to recognize their research expertise and collaborate globally provide a thoughtful conclusion. The section effectively ties back to the initial rationale, reinforcing the significance of the study in informing strategic decisions and policies at the country level.

Is the work clearly and accurately presented and does it cite the current literature?

Yes

If applicable, is the statistical analysis and its interpretation appropriate?

Yes

Are all the source data underlying the results available to ensure full reproducibility?

Yes

Is the study design appropriate and is the work technically sound?

Yes

Are the conclusions drawn adequately supported by the results?

Yes

Are sufficient details of methods and analysis provided to allow replication by others?

Yes

Reviewer Expertise:

Surgery, general surgery, surgical oncology, breast, thyroid, trauma, colorectal, medical education, curriculum, assessment

I confirm that I have read this submission and believe that I have an appropriate level of expertise to confirm that it is of an acceptable scientific standard.
